# Supplementary material for: Remodeling Lipids in the Transition from Chronic Liver Disease to Hepatocellular Carcinoma
Source: Cancers (Basel). 2020 Dec 30;13(1):88. doi: 10.3390/cancers13010088 (PMC7795670; doi:10.3390/cancers13010088)
Supplement: Supplementary file 1 [file cancers-13-00088-s001.zip › cancers-1006143-supplementary/cancers-1006143-supplementary FINAL/Supplement Tables S3,4 &Figures S1-4_12-2020 revised.docx]

**SUPPORTING INFORMATION FOR:**

**Remodeling lipids in the transition from chronic liver disease to hepatocellular carcinoma**

Israa T. Ismail ^1,2^, Ashraf Elfert ^1^, Marwa Helal ^1^, Ibrahim Salama ^1^, Hala El-Said ^1^, and Oliver Fiehn ^2^ *

1.National Liver Institute, Menoufia University, Shebeen El Kom, 55955, Egypt

2.West Coast Metabolomics Center, University of California Davis Genome Center, Davis, CA, 95616 USA

*corresponding author; E-mail: ofiehn@ucdavis.edu**.**

**Table S3. Clinical and biochemical characteristics of study subjects.**

| Parameter | HCC | Chronic Liver Disease  (CLD) | Healthy Control | P-value |
| --- | --- | --- | --- | --- |
| Number | 23 | 15 | 15 |  |
| Gender (M/F) | 17/6 | 11/4 | 10/5 | 0.877 ^(1)^ |
| Age (Years)  Mean (range) | 53 (23–73) | 46  (24–67) | 50  (23–64) | 0.311 ^(2)^ |
| Alanine transaminase (IU/L)  Mean (range) | 56  (18–160) | 58  (20–134) | 27  (16–37) | 0.878 ^(a)^  0.003 ^(2)^ |
| Aspartate transaminase (IU/L)  Mean (range) | 65  (21–195) | 48  (20–119) | 26  (18–35) | 0.115 ^(a)^  0.001 ^(2)^ |
| Hemoglobin (g/L)  Mean (range) | 12.9  (9.3–16.7) | 14  (11.9–16.6) | 13  (11.8–14.3) | 0.011 ^(a)^  0.02 ^(2)^ |
| Platelet count (10^3^/L)  Mean (range) | 193  (53–686) | 240  (172–355) | 217  (166–260) | 0.158 ^(a)^  0.370 ^(2)^ |
| International normalized ratio  Mean (range) | 1.34  (1–2.8) | 1.03  (0.9–1.14) | 0.97  (0.9–1.1) | 0.005 ^(a)^  0.001 ^(2)^ |
| current HCV infection (Y/N) ^(c)^ | 13/10 | 12/3 | 0/15 | 3.2 × 10^−5^ ^(1)^  0.13 ^(b)^ |
| current HBV infection (Y/N) ^(c)^ | 2/21 | 3/12 | 0/15 | 0.18 ^(1)^  0.25 ^(b)^ |
| Alfa fetoprotein (AFP) (ng/mL)  Mean (range) | 268.3  (2.6–2000) | 8.9  (1.53–60) | 2.6  (1.8–3.5) | 0.021 ^(a)^  0.016 ^(2)^ |
| Child Pugh score | A | No cirrhosis | **-** |  |

^(a)^ t-test between HCC and CLD patients. (b) Chi-square test between HCC and CLD patients. (c) Remaining eight HCC patients had recent HCV infection up to 2 years prior to surgery. (1) Chi-square test for healthy controls, HCC, CLD. (2) ANOVA test for healthy controls, HCC, CLD.

**Table S4. Characteristics of hepatocellular carcinoma (HCC) patients and HCC tissues**


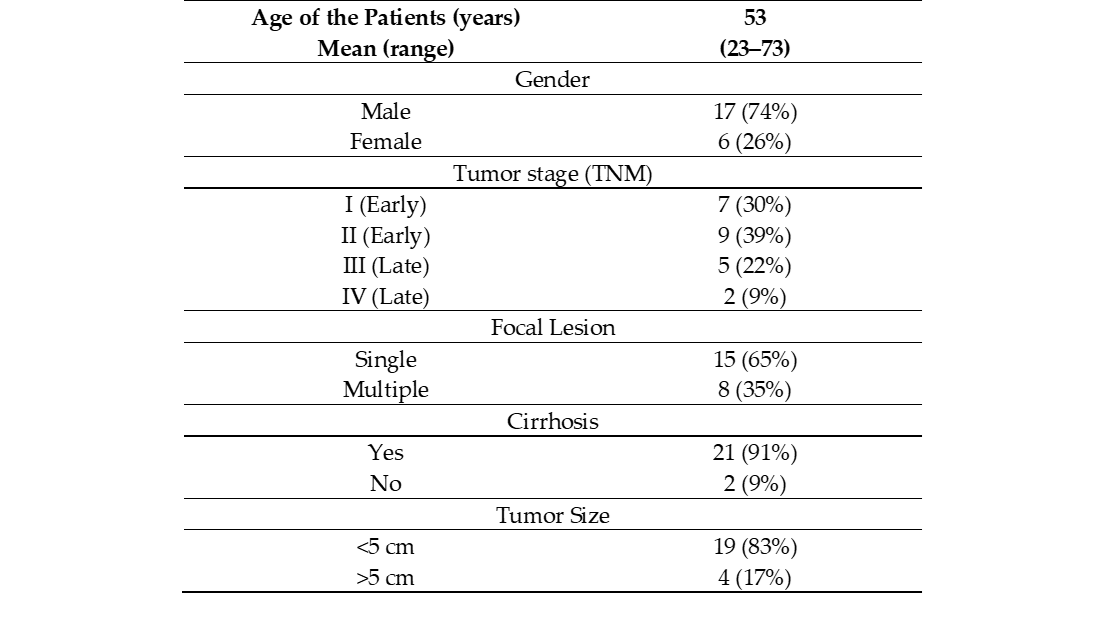


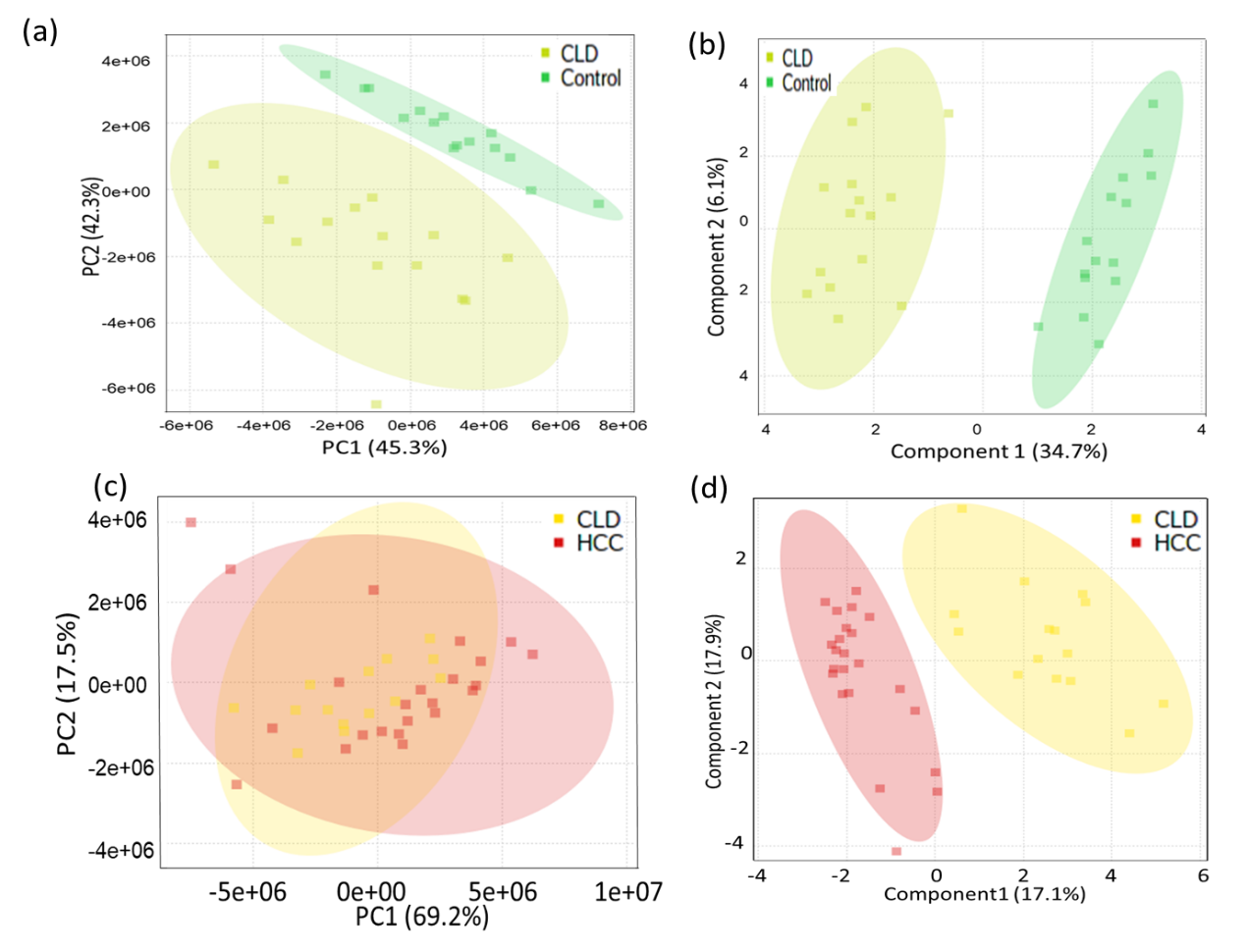


**Figure S1. Multivariate statistics comparison of blood lipidomics by unsupervised Principal Component Analysis (a,c) and by supervised sparse partial least squares discriminant analysis (b,d) .**

1. Principal component analysis of CLD patients versus healthy control subjects.
2. Sparse partial least squares discriminant analysis of CLD patients versus healthy control subjects.
3. Principal component analysis of CLD versus HCC patients
4. Sparse partial least squares discriminant analysis of CLD versus HCC patients.


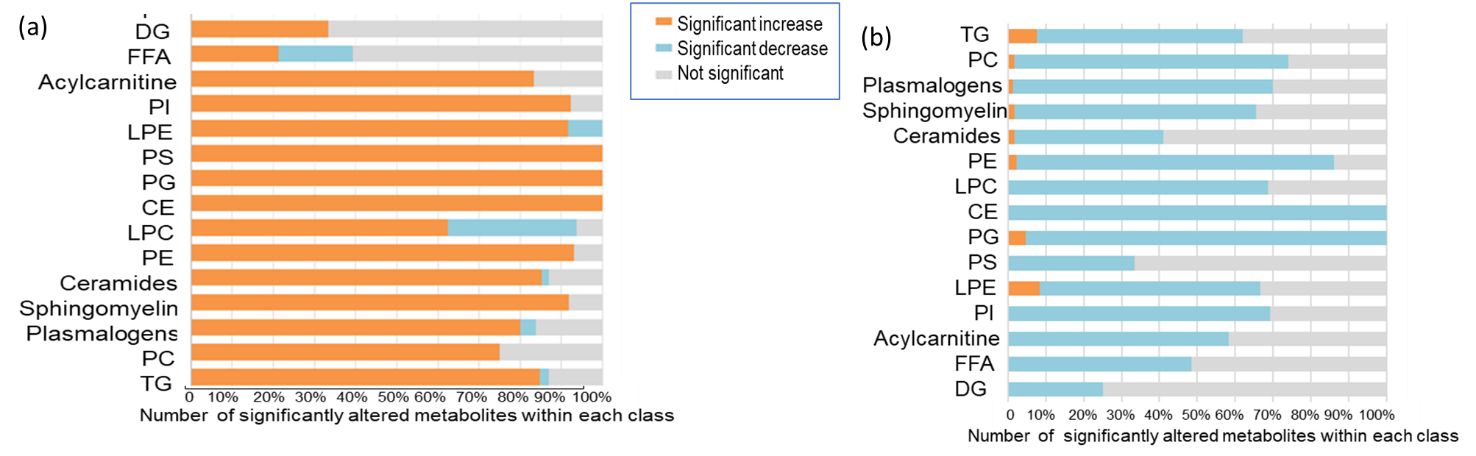


**Figure S2.** Percentage of dysregulated lipids as presented in ChemRICH in Figure 4.
Orange >1.5 fold-change increase, blue < 0.5 fold-change decrease, grey= not significantly changed

(a) Percentage of significantly altered blood lipids of CLD patients compared to healthy control subjects

(b) Percentage of significantly altered blood lipids of HCC compared to CLD patients


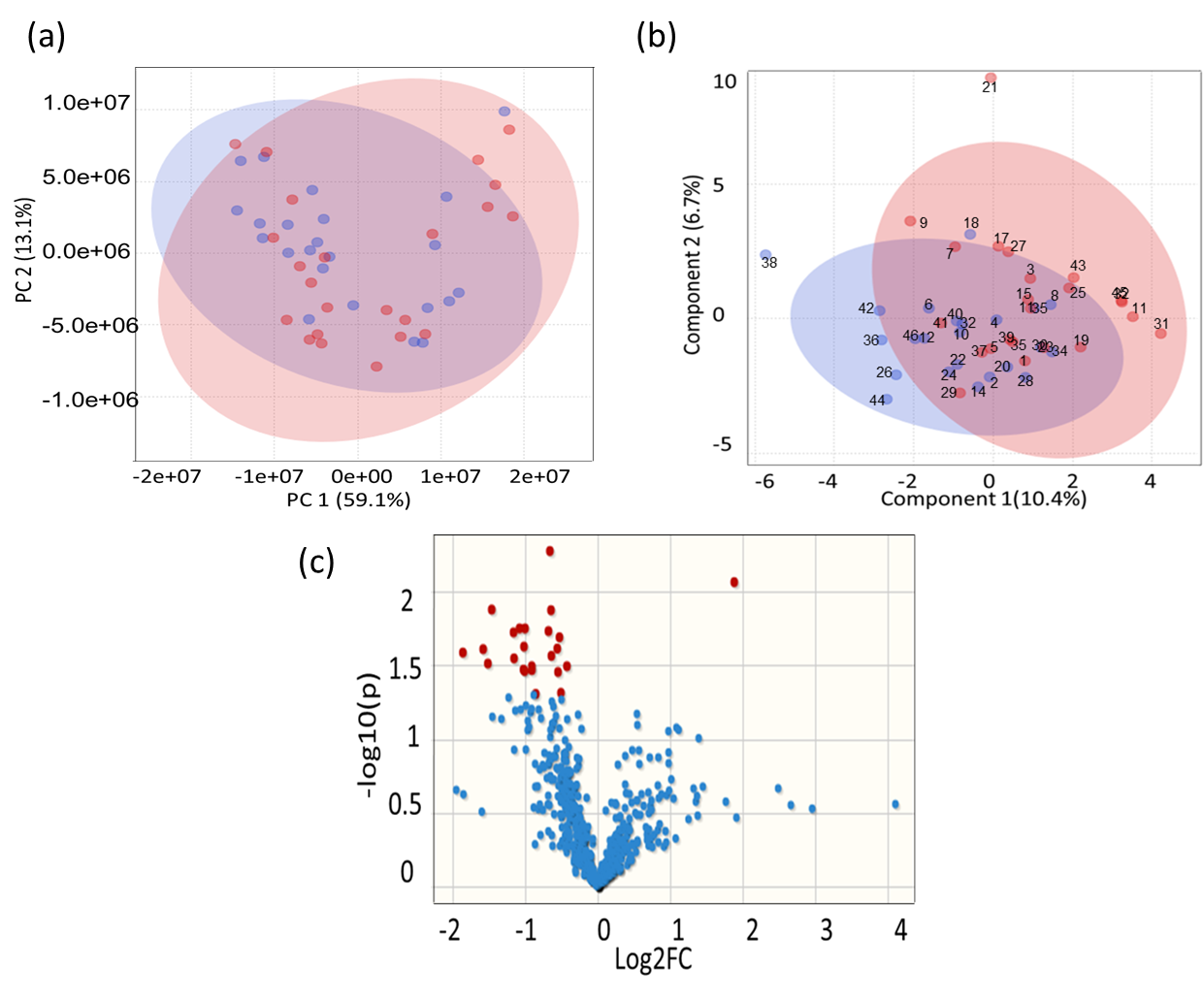


**Figure S3.** Multivariate statistics analysis of lipids in HCC tumor tissues compared to nontumor hepatic tissue. Red dots represent significantly changed lipids with univariate statistics raw *p* value < 0.05.

(a) Principal Component Analysis (PCA) of lipids in HCC tumor tissues versus non-tumor hepatic tissues.

(b) Supervised sparse Partial Least Squares Discriminant Analysis (sPLSDA) of lipids in HCC tumor tissues versus non-tumor hepatic tissues.

(b) Volcano plot of lipids in HCC tumor tissues versus non tumor hepatic tissues.


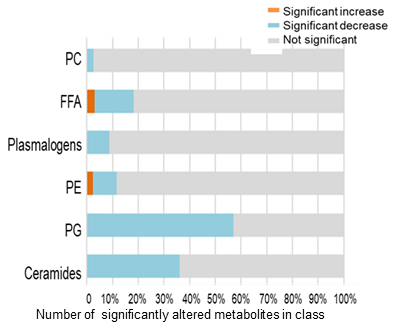


**Figure S4.** Bar chart representing percentage and direction of alteration in lipids in each significant altered class in HCC tissues versus non-tumor hepatic tissues.
